# Immune-Related Uncommon Adverse Events in Patients with Cancer Treated with Immunotherapy

**DOI:** 10.3390/diagnostics12092091

**Published:** 2022-08-29

**Authors:** Víctor Albarrán-Artahona, Juan-Carlos Laguna, Teresa Gorría, Javier Torres-Jiménez, Mariona Pascal, Laura Mezquita

**Affiliations:** 1 Medical Oncology Department, Hospital Clinic i Provincial de Barcelona, 08036 Barcelona, Spain; 2 Medical Oncology Department, MD Anderson Cancer Center, 28033 Madrid, Spain; 3 Immunology Department, Hospital Clinic i Provincial de Barcelona, 08036 Barcelona, Spain; 4 Medicine Department, University of Barcelona, 08007 Barcelona, Spain

**Keywords:** immunotherapy, immune check-point inhibitors, uncommon immune-related adverse events, diagnosis, toxicity

## Abstract

Immunotherapy has dramatically changed the therapeutic landscape of oncology, and has become standard of care in multiple cancer types in front or late lines of therapy, with some longstanding responses and outstanding results. Notwithstanding, its use has brought a totally unique spectrum of adverse events, characterized by a myriad of diverse manifestations affecting nearly every organ and system of the body, including the endocrine, nervous, cardiac, respiratory and gastrointestinal systems. Uncommon adverse events, defined as those occurring in less than 1% of patients, comprise an even more heterogeneous group of diseases that are being seen more recurrently as the use of immune check-point inhibitors increases and indications spread in different tumor types and stages. Here, we comprehensively review some uncommon, but exceedingly important, immune-related adverse events, with special emphasis in the clinical approach and diagnostic workup, aiming to reunite the evidence published previously, allowing an increase in awareness and knowledge from all specialists implicated in the diagnosis, treatment, and care of cancer patients treated with immunotherapy.

## 1. Introduction

Immunotherapy has changed the therapeutic landscape in oncology. Since the first approbation of ipilimumab in metastatic melanoma, immune check-point inhibitors (ICI) have become standard of care in multiple types of cancer in front and late lines of therapy [1]. Nevertheless, ICI have brought a completely new spectrum of side effects, commonly referred as immune-related adverse reactions (irAEs) [2]. First described in the pivotal trials of ICI, it mainly affects the endocrine, digestive, and respiratory systems, as well as the liver, joints, and skin; although, almost every organ or system in the body may be affected [3,4,5,6,7,8,9]. The increasing indications of check-point inhibitors and their widespread use have led to the description of uncommon (less than 1%), rare, and very rare irAEs—defined as those occurring at a frequency of less than 0.1 and 0.01%, respectively [10,11,12,13,14,15], and as the proportion of patients receiving immunotherapy on a routine basis exponentially rises, these previously overlooked adverse events are becoming more recurrent, as uncommon new manifestations arise and potentially relate to check-point inhibitor therapy [16]. Adverse events affecting the eye, neuromuscular junction, muscles, the hematologic system, and previously unreported endocrine glands have been described, as well as new associations with formerly idiopathic or autoimmune diseases. Moreover, the use of ICI combinations (i.e., anti-PD(L)-1 and anti-CTLA-4 antibodies) has, furthermore, increased the appearance of some infrequent irAEs. Here, we present a comprehensive review of irAEs with incidences below 1%, with the aim of highlighting their relevance and increasing the clinical suspicion to bring a prompt, accurate, and precise diagnosis, which could, subsequently, lead to adequate treatment strategies with improved survival and the least sequalae. Nonetheless, a multidisciplinary approach involving multiple medical and surgical specialties, as well as radiologists and pathologists, may all be needed to maximize the efficacy of the diagnostic workup, and when distinguishing between similar diseases with subtle disparities and completely different approaches becomes crucial to offer appropriate treatment.

**Figure 1:**
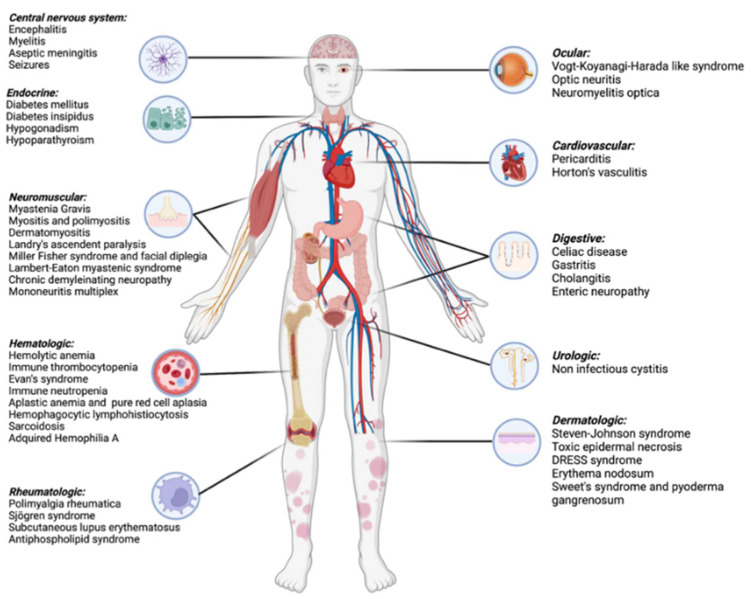
Uncommon irAEs are defined as those occurring at an incidence of less than 1%, whereas rare and very rare irAEs present at a frequency of less than 0.1 and 0.01% of cases, respectively. Other adverse reactions have a not-defined frequency, although its real incidence might be higher than previously published. Some anecdotical reports are also included here, given their rarity and, yet, interesting forms of presentation.

A schema of the most important and best-described uncommon irAEs is shown in Figure 1.

## 2. Materials and Methods

We reviewed the European Medicine Agency (EMA) product information documents [10,11,12,13,14,15] and pivotal trials of approved ICI to define the frequency of irAEs [3,4,5,6,7,8,9], selecting those classified as infrequent, rare, and very rare. Next, we analyzed published reviews and reported cases of iRAEs fulfilling these criteria, as well as those of unknown frequency using PubMed/Medline and Cochrane (in English and Spanish language) from January 2009 to June 2022. Infrequent, rare, and very rare iRAEs were defined as those presenting at a frequency of less than 1%, 0.1%, and 0.01%, respectively, according to EMA product information documents. iRAEs of unknown frequency were defined as those with an undetermined incidence on EMA product information documents, pivotal ICI trials, those published on comprehensive irAE reviews, and anecdotical case reports. We decided to focus attention on neuromuscular rather than central nervous system adverse events, given the paucity of comprehensive reviews of the former irAEs. We also focused on some poorly described, yet exceedingly interesting, manifestations, given the lack of knowledge in the description of such noteworthy diseases.

### 2.1. Neuromuscular Adverse Events

Neurological complications have been reported in 1–4.2% of patients treated with PD-1 inhibitors, which is less common compared with dermatologic, endocrine, gastrointestinal, or rheumatologic complications, reported in approximately 10–15% of PD-1 inhibitor-treated patients [17]. Neurological adverse events related to ipilimumab ranged from 3% of patients in the melanoma adjuvant trial [18] to less than 1% in other studies [3]. Neurological manifestations may be challenging and comprise a myriad of different symptoms and signs, ranging from isolated paresthesia to more complex and uncommon syndromes, requiring a high grade of suspicion and prompt recognizing, as shorts delays in diagnosis and treatment may lead to fatal outcomes. Moreover, some adverse events require specific management, and high-dose corticosteroids may not be the appropriate initial approach.

Among PD-1 inhibitor-associated neurological complications, neuromuscular disorders are the most common, accounting for 60–75% of all cases. Kao et al. reported a frequency of immune-mediated myopathies, Guillain–Barré syndrome (GBS), and myasthenia gravis of 0.15, 0.3, and 0.76%, respectively [17]. Other adverse events, such as poliradiculitis, meningo-radiculo-neuritis, and isolated neuropathies, are less common, but have also been reported [19].

#### 2.1.1. Myasthenia Gravis

Myasthenia gravis (MG) has been reported to be the most common neuromuscular joint ICI-related adverse event, although recent reports have demonstrated a lower frequency than previously described [17]. Makarious et al. published a comprehensive review of 23 reported cases, with 72.7% being de novo presentations, 18.2% exacerbations of pre-existing myasthenia gravis, and 9.1% exacerbations of subclinical myasthenia [20].

Onset can vary, ranging from 2 to 12 weeks after the onset of immunotherapy (median, 6 weeks) and a median of 2 cycles (range, 1–4 cycles) [16,21,22,23,24,25,26,27,28,29,30,31,32].

Acetylcholine receptor antibodies (AChR-Ab) may be positive in up to 2/3 of cases [17], whereas Suzuki et al. reported an equal incidence of AChR-Ab positive and negative patients, with no positivity for anti-muscle-specific kinase antibodies (aMuSK-Ab). Lipoprotein receptor-related protein-4 (LRP4) antibody positivity has not been reported in ICI-related MG.

Diagnosis is made based on clinical findings and positivity to AChR-ab, with no differences with respect to ocular, limb, and neck weakness between patients with anti-PD-1- and anti-CTLA-4-related MG. However, facial weakness and bulbar symptoms appear to be more common in anti-PD-1-related MG, as well as the severity of disease and respiratory failure. Diagnostic workup comprises electromyographic (EMG) analysis, including repetitive nerve stimulation and/or single fiber EMG, creatine kinase (CK), and troponin I levels. Electrocardiogram or Holter monitoring, echocardiogram, muscle biopsy, and muscle antibody testing may be indicated if creatine kinase levels are elevated [17,33,34]. The edrophonium test may be positive in half of patients [33]. Treatment may include pyridostigmine, high-dose IV corticosteroids, intravenous immunoglobulins (IVIG), and plasmapheresis [19,27,31]. Corticosteroids alone may not be sufficient to ameliorate symptoms, and some patients can present either progression or no improvement of symptoms during therapy [21,25,29]. ICI-related myasthenia gravis does not strictly predict good outcomes, with reports of progression both during and after disease onset [22,28,33], and mortality rates as high as 19% [31].

Patients with myasthenia gravis are at a special risk of concomitant ICI-related myopathies, which are described elsewhere in this article. Johnson et al. reported an incidence of 8.8 and 16.2% of concurrent myocarditis and myositis, respectively [31], although occurrence can rise up to 25% [33].

Although not previously associated, there have been recent reports of Lambert–Eaton myasthenic syndrome (LEMS) in the context of non-small cell lung cancer and me-lanoma immunotherapies. However, differential diagnosis between an immune-related adverse event and a late paraneoplastic manifestation of cancer remains challenging in this setting [35,36,37,38,39,40]. A summary of the published cases of ICI-related LEMS can be found in Table 1.

#### 2.1.2. Myopathies

Although previously overlooked, ICI-related myopathies are increasingly being diagnosed, and range second in frequency after myasthenia gravis. Liewluck et al. identified 5 cases of myositis among 654 patients receiving PD-1 inhibitors [34], with other authors reporting an estimated incidence of 0.7–1% [17,18]. Manifestations include polymyositis, necrotizing autoimmune myopathy, and granulomatous myositis, among others. Sheik et al. presented a single case of ipilimumab associated with an anti-Jo antibody negative dermatomyositis [41]. Time to symptom onset ranges from 2 to 9 weeks, with a median of 3 weeks [17,42].

Main symptoms include dyspnea (35%), dysphagia and dysarthria (25%), proximal or generalized limb weakness (60%), myalgia (45%), and fatigue (25%). Ocular symptoms, such as ptosis or diplopia, though seen in non ICI-related myopathies, may be present in up to 50% of anti PD-1 associated myopathies, and are increasingly becoming a distinctive feature [34]. High clinical suspicion is needed, and a careful physical examination looking for the dermatologic signs associated with dermatomyositis (heliotrope rash, Gottron papules, and flagellated erythema) is warranted [43]. CK levels are elevated in up to 43% of cases, and 30% of patients may have a concomitant ICI-related side effect at the time of presentation, mainly thyroiditis [44].

Myocarditis overlaps with myositis in 25–32% of cases, and with myasthenia gravis, in 11% [18,45], although some papers have pointed concurrent incidences as high as 45% [46], with reports of concurrence of the three entities at the same time [25,33].

Myositis antibodies are negative in most patients [17], yet striational antibody positivity has been reported in some cases. The diagnostic workout incorporates a comprehensive panel of antibodies associated with myasthenia gravis, as well as paraneoplastic and inflammatory myopathies (including anti-synthetase syndrome) [42,47,48].

Antibodies related to inflammatory myopathies are usually divided into two groups: myositis-associated antibodies and myositis-specific antibodies. The former are common in autoimmune overlap syndromes, especially systemic sclerosis (anti-Ro/SSA, anti-DNAPK, anti-PM-Scl, and anti-Scl70), and the latter includes eight antibodies directed against histidyl-transfer-RNA-synthetase (anti Jo-1), which identifies patients suffering from anti-synthetase syndrome, with unique clinical features and antibodies directed against SRP, an RNA-protein cytoplasmic complex. On the other hand, dermatomyositis is associated with a very specific subset of antibodies targeting melanoma differentiation antigen 5 (MDA5) and transcriptional intermediary factor 1 (TIF-1, as well as anti-Mi-2 and anti-NXP2 antibodies) [47,48].

Electromyography usually reports a myopathic pattern with fibrillation and myopathic recruitment. Muscle biopsies may reveal fascicular myonecrosis and phagocytosis, regeneration, mild perifascicular atrophy with increased endomysial connective tissue, and perivascular inflammatory infiltrates, mostly consisting of CD8^+^ T-cells [42,43].

Treatment strategies include the cessation of ICI administration, IVIG, or plasma exchanges, and even infliximab [17,25,34,42,46,47,49,50].

Scarce information has been found regarding outcomes in patients with ICI-related myopathies, howeverKadota et al. reported disease control in 5 cases (2 partial responses) out of 15 diagnosed with ICI-related myositis [43].

#### 2.1.3. Guillain–Barré Syndrome

Guillain–Barré Syndrome (GBS) is nowadays considered the third most common ICI-related neuromuscular complication. Reported cases include acute inflammatory demyelinating polyradiculoneuropathy (formerly known as Landry’s ascendent paralysis), Miller Fisher spectrum GBS, and acute motor and sensory axonal neuropathy (AMSAN), with an estimated incidence of 0.2 to 0.3% [16,17,19]. Onset can vary, with a range from 4 to 68 weeks after anti-PD-1 initiation, although early occurrence after only 1 dose of ICI has been reported [51,52], mostly related to a combination of PD-1 and CTLA-4 inhibitors, which accounts for almost 25% of the cases [52].

The clinical presentation is similar to non-immune related GBS, with ascending limbs weakness of subacute onset with hyporeflexia and numbness being the hallmark of the disease. Hence, a high grade of suspicion is necessary for a prompt diagnosis and appropriate treatment, especially in the initial phases and when matching symptoms and signs that may be otherwise overlooked. This is of utmost importance when it comes to GBS variants, such as Miller Fisher syndrome or the bilateral facial weakness variant [53]. Electromyogram findings of acute demyelinating polyradiculoneuropathy (prolonged distal latencies, low conduction velocities), as well as cerebrospinal fluid alterations, such as mild pleocytosis with lymphocytic predominance -in up to half of cases- and hyperproteinorrhaquia and albumincytologic dissociation in 44% of patients might help in the diagnosis. Anti-gangliosides antibodies have occasionally been found positive [54].

Differential diagnosis includes meningeal carcinomatosis, botulism, tick paralysis, and Lyme disease [51]. Dorsal midbrain syndrome (Parinaud’s syndrome) and other causes of total ophthalmoplegia must also be taken into account when the Miller Fisher variant is being considered. The diagnostic workup should comprise HIV and hepatitis A, B, C, and E antibodies; a feces culture for *Campylobacter jejuni* detection; *Mycoplasma pneumoniae*; cytomegalovirus (CMV); and Epstein–Barr (EBV) and Zika virus serologies or PCR detection in cerebrospinal fluid, as well as Gram stain, bacterial culture, and cytology [53,54]. Testing for anti-onconeural antibodies (anti-Hu, anti-Ri, anti-Yo, anti-CV2, and anti-Tr, among others) and a brain MRI are also necessary to rule out paraneoplastic syndromes [55] and brain/meningeal metastases, respectively [55,56].

Therapeutic strategies can vary, being IVIG and plasma exchange the treatment cornerstone, yet some reports point to high-dose IV corticosteroids, which are usually spared in non-ICI-related Guillain–Barré syndrome [17,57,58].

Despite a prompt diagnosis and treatment initiation, Guillain–Barré syndrome portends poor prognosis, with mortality rates ranging from 10 to 22% [31,52]. To our knowledge, no information regarding outcomes in patients with cancer- and ICI-related GBS has been published.

A summary of the diagnostic workup of the neuromuscular irAE’s can be found in Table 2.

### 2.2. Hematological Immune Related Adverse Events

Hematological toxicities from ICI are among the least described immunotherapy adverse events. Although the incidence in most series is below 1% [59,60], their occurrence is increasing with the widespread use of ICI across multiple types of solid tumors. However, they have not been extensively characterized so far.

Hematological irAEs encompass a wide spectrum of different manifestations with great diagnostic difficulties, often derived from multicausality, idiosyncrasy, and a lack of specific biomarkers for accurate diagnosis. Thus, most of associations are concluded as “probable” or “possible”, making extremely difficult to establish a definite relation [61]. Hematological irAEs tend to occur early in treatment, with a median onset of 40 days (range 3–405 days), and mortality rates ranging from 12 to 34% [59,62].

#### 2.2.1. Immune Cytopenias

Various types of cytopenias have been described related to ICI, including immune thrombocytopenia, autoimmune hemolytic anemia (AIHA), neutropenia, and aplastic anaemia [60].

Immune thrombocytopenia is, by far, the most common hematological irAE, followed by hemolytic and aplastic anemias [61]. The median time to onset of ICI-induced immune thrombocytopenia is 70 days (range, 12 to 173 days), with most cases occurring in the first 12 weeks after ICI initiation, and an average platelet count of around 60,000/uL (range, <5000 to 104,000/uL) and relative decreases of 70% from baseline (range, 38 to 99%) [60].

The severity can be assessed using the bleeding score proposed by Khellaf et al., which takes into account age, type and location of bleeding by organs. According to it, patients with platelet levels less than 20 × 10^9^/L and a bleeding score of >8 points may warrant immediate treatment with IVIG to avoid complications [63].

Non-ICI related immune thrombocytopenia is a diagnosis of exclusion, and most cases are idiopathic in etiology. Thus, a definite diagnosis may be challenging in the context of cancer immunotherapy, given the lack of specific testing and wide differential diagnosis.

Autoimmune hemolytic anemia (AIHA) occurs at a variable frequency, and it seems to be slightly more common with PD-1/PD-L1 inhibitors [62] The median time to onset is about 50 days, and mortality is nearly 15%. Some reports point to an association between ICI-induced AHAI and chronic lymphocytic leukemia, sugges-ting a potential increased risk of hematological immune toxicity in these patients [64]. Delanoy et al. published a cohort of 35 cases (3 with chronic lymphocytic leukemia) and suggested to rule out the presence of an underlying chronic lymphocytic leukemia in any patient with a suspected immune-related hematological toxicity.

Autoimmune anemia is characterized by a positive serum direct antiglobulin test (DAT) by anti-IgG/IgM or anti-IgG/IgA and/or an anti-C3d, and the depiction of the thermal optimum reaction distinguishes between cold (reacts maximally at 4 °C) or warm (reacts maximally at 37 °C) antibodies, which is considered essential when deciding the the-rapeutic approach in AIHA. In non-ICI related AIHA, cold antibodies (usually an IgM with a C3-type positive DAT) can be due to a viral (e.g., CMV or EBV) or bacterial (e.g., *Mycoplasma pneumoniae*) infections. In other cases, the underlying disease is a low-grade B-cell lymphoproliferation with an IgM kappa monoclonal gammopathy, also known as cold agglutinin disease [65]. Warm antibodies are generally associated with an autoimmune condition (e.g., systemic lupus erythematosus) or an underlying lymphoid malignancy (e.g., chronic lymphocytic leukemia or non-Hodgkin B-cell marginal zone lymphoma) [66].

A direct Coombs test has been reported to be positive in nearly 100% of ICI-related AHAI, with up to 30% of patients demonstrating the presence of cold antibodies or cold agglutinins [59].

The concurrence of both immune thrombocytopenia and AHAI—classically known as Evans Syndrome—has also been reported, and the diagnosis of one of the diseases should lead to actively discard the other as part of the workup [62]. There have been few reports of ICI-related cryoglobulinemia; one of them was also associated with cold agglutinin disease [67,68,69]. On the contrary, no reports of ICI-related biphasic hemolytic anemia (also known as Donath Landsteiner disease) have been found.

Immune neutropenia appears to be rare and occurs mostly in combination with anemia. Nevertheless, its occurrence is usually severe and prolonged, with blood neutrophils counts near 0/ul and a median duration of 16 days. Concomitant infections occur in almost 50% of cases. Neutropenia can be associated with the large granular lymphocytes syndrome, which must be assessed in blood and bone marrow if the count is >0.5 × 10^9^/L by T and NK lymphocyte immunophenotyping [66]. Positivity to antineutrophil membrane antibodies or antineutrophil cytoplasmic antibodies (ANCA) have been demonstrated in some cases of non-ICI related immune neutropenia, although its estimated frequency has not been reported [70,71,72,73].

Pancytopenia occurring after ICI administration should raise concern about aplastic anemia. Cytopenias are often prolonged with the subsequential need for transfusion support and bleeding and infection risks, and resolution is rare. The severity of the bone marrow failure can be assessed with the modified Camitta’s criteria, which classifies aplastic anemia into three categories (severe, very severe, or non-severe) according to bone marrow cellularity, peripheral blood cell levels, and reticulocyte count [74]. The possibility of paroxysmal nocturnal hemoglobinuria should always be considered, although no reports of ICI-related paroxysmal nocturnal hemoglobinuria have been found.

Hypoproliferative anemia resembling pure red cell aplasia (PRCA) has also been reported [59,62,75], alone or in combinations with AIHA, with reports showing variable onset times (between 9 and 93 weeks) [76]. It is characterized by normocytic, normochromic anemia, with reticulocytopenia and the absence of erythroid precursors in an otherwise normal bone marrow. PCRA can be related to infective diseases such as parvovirus B19, drugs such as azathioprine and procainamide, or even autoimmune and neoplastic di-seases such as thymoma or lymphoma. Antibodies to both erythroid progenitors and erythropoietin have been detected in some cases [77].

Increased levels of eosinophils have also been described. Nonetheless, it is usually considered a benign finding with no specific treatment needed. Some authors have related eosinophils early dynamic changes and counts as a predictor of long-term disease control in metastatic melanoma [78,79] and non-small cell lung carcinoma [80], and ICI treatment should not be interrupted [81,82]. In patients with overt hypereosinophilia (>1500 cells/mm^3^), a diagnostic workup including allergic, infective, paraneoplastic, and primary hematological diseases should be performed. DRESS syndrome, a rare and potentially fatal dermatologic complication also associated with ICI, must be considered, and is described elsewhere in this article.

Hematological and tumor-related causes of cytopenias (such as bone marrow malignant infiltration) must be comprehensively ruled out, as well as parvovirus B19 infection. Recent medical history should be screened for other possibly implicated medications such as cotrimoxazole, clozapine, propylthiouracil, penicillin, rituximab, flecainide, metformin, and colchicine among others [61].

Relevant diagnostic tests include a peripheral blood smear; reticulocyte count; hemolysis assays (lactate dehydrogenase, haptoglobin, and bilirubin); and iron, folate, and vitamin B12 levels to rule out megaloblastic anemia. Measurement of antinuclear antibodies (ANA) in serum might be helpful in ICI-induced immune thrombocytopenia, as it renders positive in up to 33% of patients [59].

Bone marrow analysis has been performed in the majority of cases published by Omar et al., to determine whether a central or peripheral cause of cytopenia is present [61], and it is recommended if ancillary tests point to hypoproliferative anemia or bicitopenia, although it is not mandatory for the diagnosis of AIHA in the presence of a positive direct antiglobulin test (Coomb’s test). The main bone marrow features of immune-related cytopenias are listed in Table 3 [75,81].

A transfusion of pre-heated red blood cells for cold antibody AIHA and folic acid supplementation should be given to all symptomatic patients. In hemolytic anemia induced by cold antibodies, corticosteroids are notoriously ineffective. Treatment is based on rituximab, eventually combined with chemotherapy with an alkylating agent. The treatment of ICI-related aplastic anemia relies on corticosteroids, granulocyte-colony stimulating factor (G-CSF), or even immunosuppressive agents. The use of the thrombopoietin receptor agonist (eltrombopag) should be evaluated individually [84].

Immune neutropenia requires special consideration, as treatment with corticoste-roids may enhance immunosuppression, and a therapeutic approach with G-CSF and prophylactic antibiotics to prevent opportunistic infections can be recommended [59].

There is no comprehensive review regarding outcomes in patients with cancer- and ICI-related cytopenias, although Wright and Brown reported a patient treated with nivolumab for refractory primary mediastinal B-cell lymphoma who achieved a complete response coinciding with a ICI-induced severe neutropenia [71].

#### 2.2.2. Hemophagocytic Lymphohistiocytosis

Hemophagocytic lymphohistiocytosis (HLH) is an uncommon disease characterized by hyperactivation of the immune system and progressive organ disfunction [85,86,87]. HLH is divided into primary (genetic) and secondary (acquired) [86], with the latter being triggered by viral infections (EBV, Dengue virus, HIV virus, CMV, hepatitis A), bacteria (*Rikettsia* spp., tuberculosis), parasites and fungi (*Histoplasma* spp., *Leishmania* spp., *Plasmodium* spp., *Toxoplasma, P. jiroveci, Aspergillus* spp.), autoimmune disorders such as systemic lupus erythematosus and adult-onset Still disease, solid tumors, and drugs [86,88].

Of all hematological iRAEs, hemophagocytic lymphohistiocytosis appears to be the most life-threatening condition, with mortality rates of nearly 50%. Likewise, it is certainly one of the rarest, with some reports suggesting an incidence of less than 1% [89].

Median time to onset is reported to be about 6.7 weeks (range 2.9–15.4 weeks), although Noseda et al. reported 11% of HLH occurring after a single dose of ICI. There appears to be a clear male-to-female predominance (2 to 3:1) [90].

The diagnostic workup should include all the ancillary tests necessary to fulfill the HLH-2004 revised diagnostic criteria [85], including fever, splenomegaly, cytopenias, hypertrygliceridemia of hypofibrinogenemia, evidence of hemophagocytosis in bone marrow or other lymphoid organs, elevated ferritin and CD25 levels, and no evidence of neo-plasia), although other authors recommended the use of the H-score, as it may be more useful in diagnosing secondary HLH in adults [91]. A comprehensive panel to rule out triggering or concomitant infective and autoimmune diseases must always be performed.

Other abnormal clinical and laboratory findings consistent with the diagnosis are: cerebromeningeal symptoms, lymph node enlargement, jaundice, edema, skin rash, hepatic enzyme abnormalities, hypoproteinemia, hyponatremia, high VLDL and low HDL levels.

Bone marrow may demonstrate a normoblastic cellularity with hemophagocytic macrophages, and it must rule out neoplastic myelopthisis [89]. If the bone marrow study is negative or inconclusive and the clinical suspicion is still high, biopsies can be obtained from other organs. Serial marrow aspirates may also be indicated for an accurate diagnosis.

Differential diagnosis is broad and includes sepsis, hepatitis, SARS-Cov-2 infection, DRESS syndrome, and adult-onset Still’s disease, among others.

Oncologic outcome has not been comprehensively addressed, yet Mizuta et al. showed disease control in 6 out of 7 melanoma patients (three complete responses and three partial responses) [91]. Similarly, Lorenz et al. reported a complete response in a patient with a metastatic prostate cancer after 9 months of treatment with pembrolizumab who developed an HLH and successfully recovered following high-dose corticosteroids, plasma exchanges, etoposide, and tacrolimus [92], whereas Olivares-Hernández et al. successfully treated a patient with metastatic choroidal melanoma and an ipilimumab-induced HLH with the anti-interleukin-6 receptor antibody, tocilizumab [93].

#### 2.2.3. Sarcoidosis-Like Reaction

Drug-induced sarcoidosis or sarcoidosis-like reaction (SLR) have been described with ICI. SLR is a multisystemic granulomatous reaction that occurs after exposure to a drug, in the absence of history suggestive of the disease prior to treatment initiation, which ultimately improves after its withdrawal. Incidence is very low [94], with some authors reporting a frequency of 0.2% [95], mostly associated with anti CTLA-4 antibo-dies, although several cases have been reported with anti PD-1 antibodies and ICI combinations [96]. The time between ICI starting and SLR diagnosis ranges widely, from 3 weeks to almost 2 years, with a median onset time of 4–5 months [96].

Clinically, SLR is similar to non-ICI related sarcoidosis, with patients typically experiencing cough, arthralgia, and skin lesions. Other authors have reported uveitis, hypercalcemia, hypersplenism, nephritis [97], pituitary granulomas [98], polyneuropathy, or elevated angiotensin-converting enzyme levels [99]. Bilateral pulmonary hilar lymphadenopathies are the most frequent findings on CT scan. Histopathologically, SLR shows noncaseating epithelioid giant-cell granulomas surrounded by lymphocytes, with occasional birefringent foreign bodies, asteroid bodies, and Schaumann bodies, also indistinguishable from classic sarcoidosis [100].

Diagnosis is only established once alternative causes of granulomatous diseases (e.g., infection, inflammatory disorders, malignancies) have been reasonably excluded. Differential diagnosis encompasses special complexity in lung cancer patients, in whom, the enlargement of mediastinal and hilar nodes can also suggest progressive or relapsing disease [94]. Histological confirmation is highly recommended to differentiate SLR from alternative causes. Management is controversial, as it usually improves upon the discontinuation of ICI and/or corticosteroid treatment. The diagnosis of SLR does not universally mandate specific therapy, unless it is associated with significant symptoms or organ dysfunction, and active surveillance may be indicated in mild cases [101].

#### 2.2.4. Bleeding Disorders

Acquired hemophilia A has been reported in a melanoma patient after three cycles of ipilimumab completely recovered after treatment with corticosteroids and recombinant VIII factor [102], and in two patients with non-small cell lung carcinoma on nivolumab successfully treated with cyclophosphamide [103] and rituximab [104]. To our knowledge, no cases of ICI-related acquired Von Willebrand’s syndrome or other bleeding disorders have been reported.

### 2.3. Immune-Related Endocrinopathies

Immune-related endocrinopathies from ICI include thyroiditis, hypophysitis, type 1 diabetes mellitus, and adrenal insufficiency, among others [105,106]. The most common immune-related endocrinopathies are thyroid gland disorders, usually hypothyroidism, often preceded by transient thyroiditis-induced thyrotoxicosis [107]. Importantly, endocrinopathies usually require lifelong hormonal replacement, and also require adequate diagnosis, since most of symptoms are non-specific and similar to cancer progression or chemotherapy toxicities [108]. Here, we review some of the more uncommon immune-related endocrinopathies.

#### 2.3.1. Type 1 Diabetes Mellitus

ICI-related type 1 diabetes mellitus is caused by dysfunction of pancreatic islet beta cells, generating an insulin-deficient diabetes [109,110]. Immune-related diabetes mellitus occurs at a low frequency (<1%) and it appears to be more common with anti-PD-1/PD-L1 antibodies than with anti-CTLA-4 antibodies [111,112]. The onset time ranges from a few weeks, sometimes occurring after the first or second ICI administration, up to more than one year after treatment initiation [106].

The diagnosis is suspected when symptoms of hyperglycemia appear, and fasting blood sugar or random blood sugar levels exceed 126 and 200 mg/mL, respectively. Dry mouth, polydipsia and polyuria are present in mild to moderate cases, whereas general fatigue, ketoacidosis, disturbed consciousness, or coma may be present in severe cases [113,114]. In up to 70% of patients, an acute-onset ketoacidosis is the first manifestation of immune-related diabetes mellitus [106]. Fulminant type I diabetes—defined as acute-onset hyperglycemia with ketoacidosis, near-normal haemoglobin-A1c (HbA1c) levels, and the complete absence of insulin secretion—can worsen rapidly and prove fatal [114].

HbA1c is elevated in most cases, but its sensitivity for detecting ICI-related diabetes mellitus is low given the rapid time course of development [106,115], with mean levels of 7.4%. C-peptide levels, used to measure endogenous insulin secretion, are usually low. Serum antibodies are positive in 50% of patients with ICI-related diabetes mellitus. Antibodies directed to glutamate decarboxylase (Anti-GAD), islet antigen 2 (IA2), insulin (IAA), islet cell, and zinc transporter 8 (ZnT8) have all been described in patients with ICI-related diabetes mellitus [106].

Insulin therapy is the mainstay of treatment, and immediate treatment must be initiated [116]. If ketosis or ketoacidosis is present, immediate-acting insulin should be continuously administered, along with intravenous saline infusion and electrolyte replacement.

#### 2.3.2. Hypoparathyroidism

ICI-related hypoparathyroidism is considered an exceedingly rare event with few cases published [117], although there have been increasing reports of immunotherapy-related hypocalcemia, either with monotherapies and combinations [118], mainly in melanoma and non-small cell lung cancer. Yet, its frequency has not been systematically addressed. Win et al. reported a case of nivolumab/ipilimumab-induced hypocalcemia with undetectable plasma intact parathyroid hormone (PTH) levels, who also experienced immune-related thyroiditis shortly after admission. The time to onset was 1.5 months, and resolved with calcium and magnesium reposition, as well as thyroid hormone replacement and calcitriol [119]. Mahmood et al., on the other hand, reported a single case of pembrolizumab-associated hypoparathyroidism after 15 months of treatment [120].

The clinical picture may vary from asymptomatic cases to a life-threatening condition [121]. Classically-described Chvostek and Trousseau’s signs can be found if examined carefully, as typical electrocardiogram abnormalities (i.e., prolonged corrected QT interval) [122]. Acute hypocalcemia can be a medical emergency presenting with neuromuscular irritability and seizures [118], requiring a rapid diagnosis and treatment.

Antibodies directed to the calcium-sensing receptor (CaSR) have also been reported, and if yield positive, the diagnostic workup must include an antibody panel to discard the presence of a type-1 autoimmune polyglandular syndrome (APS1) [123,124]. Hypoparathyroidism should be distinguished from pseudohypoparathyroidism or hypocalcemia secondary to hypomagnesemia, which can also be accompanied by low serum calcium levels. However, these can be distinguished by PTH levels—high in pseudohypoparathyroidism, and low in hypomagnesemia [125,126]. Therefore, hypocalcemia should be thoroughly investigated before attributing it to an ICI-related hypoparathyroidism [127]

Outcomes vary on different reports, with some authors reporting long-lasting responses, as well as disease progression [128]. None of the cases published have required ICI-withdrawal, excepting those who had other concomitant iRAEs.

#### 2.3.3. Other Endocrinopathies

Central diabetes insipidus has also been described with anti CTLA-4 antibodies, mostly in the context of ICI-related hypophysitis [129]. Bai et al. reported an incidence of 1.4% of anterior hypophysitis-associated central diabetes insipidus; however, it has also been encountered as an isolated adverse event with anti-PD(L)-1antibodies, and combination therapies [130]. Bernabei et al. published a comprehensive review of 11 cases of ICI-related central diabetes insipidus, and reported a clear predominance of males (10 out of 11) and a median onset time of 112 days [131,132]. As in non ICI-related diabetes insipidus, desmopressin is the mainstay of treatment, and produces a rapid amelioration of symptoms [133,134,135]. Thsuma et al. reported a case of hypothalamitis in a stage IV bladder cancer patient after 12 cycles of atezolizumab who presented with panhypopituitarism, as well as signs of hypothalamic disfunction (i.e., severe sleep apnea and temperature dysregulation). A hypothalamic mass was detected on brain images, with complete resolution after a short trial of high-dose dexamethasone and atezolizumab withdrawal [136]. Likewise, Lupu et al. reported a case of transient pituitary ACTH-dependent Cushing syndrome after three cycles of nivolumab and ipilimumab combination for stage IV melanoma in the context of an immune-related hypophysitis, evolving towards a ACTH deficiency and, consequently, adrenal insufficiency [137]. In the last two cases, a long-lasting response was achieved.

Hypogonadism constitutes a very rare, but potentially important iRAE; however, little is known about the effect of ICI on gonadal function [138]. Secondary hypogonadism (hypogonadotropic hypogonadism) can arise in the context of ICI-induced panhypopituitarism; however, the levels of follicle-stimulating hormone (FSH) and luteinizing hormone (LH) are rarely assessed, and supplementation with gonadotropic hormones is seldom prescribed, a matter of special concern in pre-menopausal women to avoid the risk of osteoporosis, and in reproductive-age individuals, given the potential quality-of-life deterioration [139]. Albarel et al. reported gonadotroph deficiency in 11 out of 15 patients (85.7%) diagnosed with an ipilimumab-related hypophysitis. Besides central hypogonadism, direct gonadal inflammation has also been reported in association with ICI. Brunet-Possenti et al. reported a case of a 54-year-old man with a stage IV melanoma who developed transient bilateral orchitis after the first infusion of nivolumab and ipilimumab [140], and Quach et al. reported a single case of epididymo-orchitis and encephalitis after three doses of pembrolizumab for metastatic melanoma, rapidly responding to corticosteroids [141]. Testosterone levels returned rapidly to normal in the first case, whereas the latter reported no information regarding the hormonal status. Moreover, an association with impaired spermatogenesis in melanoma patients treated with ICI has recently been des-cribed [142].

### 2.4. Dermatologic Immune-Related Adverse Events

Cutaneous irAEs represent a heterogenous group of inflammatory responses to ICI that usually occur in a self-limiting time and often appear the earliest after treatment initiation (approximately 3–6 weeks) [143]. The most common are nonspecific maculopapular rash, pruritus, vitiligo, and lichenoid dermatoses, among others [144]. Nevertheless, there are some severe complications that require immediate diagnosis and treatment, such as Steven Johnson syndrome (SJS), toxic epidermal necrolysis (TEN), and drug reaction with eosinophilia and systemic symptoms (DRESS syndrome).

Most relevant uncommon cutaneous irAEs are summarized in Table 4.

#### 2.4.1. Steven Johnson Syndrome and Toxic Epidermal Necrolysis

SJS and TEN are severe mucocutaneous drug reactions which are clinically similar, except for their distribution and extension of body surface area (BSA) affected: <10% vs. >30% in SJS and TEN, respectively [165]. When extension oscillates between 10–30% of BSA, it is considered as an overlap, being defined as a part of the same disease spectrum.

The median onset time is about 25.5 days [166], ranging from 15 to 90 days of treatment initiation [145,146,167]. Initially, patients present with fever, headache, cough, and/or sore throat. Next, target-like macules appear abruptly on the face, neck, and trunk. Lesions appear simultaneously on other parts of the body, coalesce to form flaccid and sometimes painful blisters, and slough off over a period of 1 to 3 days. Palms and soles may be affected. Most patients have mucosal involvement, with two or more mucosal surfaces being involved in up to 80% of cases [168]; thus, mucositis and ulceration occur in almost 100% of cases, and gynecological involvement in females appears in approximately 77% of cases [169]. Moreover, respiratory failure may indicate a compromise of the epithelial respiratory tract.

A careful evaluation of skin lesions and the calculation of affected BSA is mandatory to determine the extension of disease. Nikolsky and Asboe-Hansen signs are both characteristic. The former consists of the dislodgment of the normal epidermis and extension of an intact blister when lateral pressure is applied on the border, whereas the latter refers to the extension of a blister to adjacent unblistered skin when pressure is put on the top of the bulla [170]; therefore, diagnosis relies primarily on characteristic history and physical examination [171,172]. Skin biopsies may show an accumulation of CD8þ cells at the dermoepidermal junction, along with keratinocyte apoptosis and PD-1 expression on skin-infiltrating T-cells and keratinocytes [173], a potentially helpful finding in cases where diagnosis is unclear. Differential diagnosis must be performed with other forms of drug-induced erythema multiforme or other bullous disorders, such as bullous pemphigoid.

Molina et al. described an unusual SJS/TEN-like reaction characterized by a generalized bullous mucocutaneous eruption that mimicked those diseases. This progressive immunotherapy-related mucocutaneous eruption (PIRME) shares some clinical features, although with a benign and favorable clinical course, good response to treatment, and delayed onset [174].

The management of these reactions requires hospitalization, intravenous corticosteroids, fluid and electrolyte management, serial dermatologic examination, and immediate ICI withdrawal due to its high mortality rate—around 37% [166], similar to non-immune-related SJS/TEN. A poor response to corticosteroids has been also reported, and other immunosuppressive therapies, such as cyclosporine or intravenous immunoglobulin, may be needed for disease control [167].

#### 2.4.2. Drug Reaction with Eosinophilia and Systemic Symptoms (DRESS Syndrome)

DRESS syndrome is a severe adverse drug-induced reaction and potentially life-threatening disease characterized by a generalized cutaneous rash, fever, eosinophilia, or atypical lymphocytes in blood tests, and systemic involvement, with the liver being the most affected organ. Symptoms begin in a range of 2–8 weeks after ICI initiation [147].

Most cases reported to date have been related to the combination of PD-1 and CTLA-4 inhibitors, but also with single agents [148,149,150]. Patients present initially with fever, malaise, headache, cough and delirium. Concomitantly, or shortly after, a diffuse and non-itchy morbilliform maculo-papular rash with neither splits nor mucosal involvement develops. Blood hypereosinophilia was present in the three cases described [148,149,150], with two out of three developing acute kidney disease, and one out of three with altered liver function tests and troponin levels. After withdrawal of ICI and intravenous corticosteroids administration, complete resolution of DRESS syndrome was achieved in all cases.

The diagnosis of the DRESS syndrome relies on accurate clinical history, physical examination, and use of the international Registry of Severe Cutaneous Adverse Reaction (RegiSCAR) group criteria for hospitalized patients with a drug rash [175]. This score includes seven items comprising fever (>38.5 °C), enlarged lymph nodes, atypical lymphocytes and eosinophilia, skin rash extension, internal organ involvement, resolution in ≥15 days, and an evaluation of other potential causes, and classifies suspected cases as definite (≥6 points), probable (4–5 points), possible (2–3 points), and discarded (<2). Nonetheless, it is mandatory to rule out other diseases, such as SJS, TEN, acute generalized exanthematous pustulosis, and other drug-related erythema multiforme.

The mortality rate is about 5–10% [176], with no specific data available for immune-related DRESS syndrome.

#### 2.4.3. Erythema Nodosum-Like Panniculitis

Erythema nodosum (EN) is the most common form of panniculitis. Its etiology is unknown, although it appears to be a hypersensitivity response to multiple antigenic stimuli, with about 30–50% of cases being idiopathic. Commonly, patients present with tender, warm, and painful erythematous subcutaneous nodules that are usually located symmetrically on pretibial areas, sometimes associated with systemic symptoms, such as fever, malaise or headache [177].

Two series of cases of ICI-related EN have been described in the literature, compri-sing four cases in total [151,152]. Three out of four patients received the combination of PD-1 plus CTLA-4 inhibitors, whereas the other was under pembrolizumab monotherapy. The onset of symptoms ranged from 7 weeks to 19 months. Patients showed painful and swelling erythematous nodules in lower extremities. Physical examination was similar in all patients, with several red tender nodules in both legs. Diagnosis was made based on an accurate clinical history, as well as physical examination with the cha-racteristic findings. In addition, a biopsy was performed with evidence of an inflammatory infiltrate composed of lymphocytes, histiocytes and occasional neutrophils or eosinophils. Immunohistochemical analysis of one of the cases showed a predominance of *CD3*+ T-cells with a CD4:CD8 ratio of ~2:1 and mostly preserved CD7 expression. CD4 and CD123 highlighted additional histiocytes and multi-nucleated giant cells, and isolated dendritic cells, respectively.

Histopathology is mandatory, and the prototypic septal panniculitis with and inflammatory infiltrate is required for proper diagnosis [178]. Furthermore, this allows differential diagnosis with other forms of panniculitis, such as eosinophilic or pancreatitis panniculitis, urticaria, rheumatoid nodules, *erythema induratum*, and other skin lesions with similar clinical appearance. Of note, most of the lesions resolve spontaneously, but if any cause is identified, specific treatment of the underlying condition invariably leads to the resolution of the associated EN [177,178].

The therapeutic approach is defined according to symptom severity, from no treatment, to treatment with topic or oral corticosteroids, minocycline and clobetasol. A close follow-up is recommended because severe relapses have been des-cribed after ICI reintroduction [178].

### 2.5. Digestive Immune-Related Adverse Events

Digestive disorders are common irAEs described with the use of ICI, especially with CTLA-4 antibodies, including diarrhea and colitis (27–54% and 8–22%, respectively), he-patitis (5–10% with monotherapy, 25–30% in combinations), and pancreatitis (0.3–3.9%) [179] as the most frequently observed, but, potentially, all the gastrointestinal (GI) tract can be affected. We will be focus on the uncommon digestive irAEs.

#### 2.5.1. Celiac Disease

Celiac disease (CeD) or gluten-induced enteropathy is an autoimmune condition in which genetically predisposed individuals experience symptoms resulting from intestinal villi damage of the duodenum and proximal small bowel triggered by gluten consumption [180].

The incidence of ICI-related CeD is unknown. Since the first case reporting a CeD related to ipilimumab published by Gentile et al. in 2013 [181], several cases have been reported with PD-L1 and CTLA-‘4 inhibitors in monotherapy and also in combination [182]. The largest series was published by Badran et al. who described eight cases of immune-related CeD [183], showing that the ICI-induced CeD had increased intraepithelial CD3^+^ and CD8^+^T cells and γδ T cells compared with conventional CeD. Interestingly, in genetically susceptible individuals, ICI exposition would promote self-reactive CD4+ T cell expansion and subsequent CD8+ T cell-induced tissue destruction [184].

Due to the low frequency of ICI-related CeD, it is often missed from the diagnostic spectrum, consequently with substantial treatment delays. Clinical manifestations vary, and include diarrhea, fatigue, malabsorption leading to weight loss, anemia, and osteoporosis [180] with a variable onset (median time to develop intestinal symptoms of 48 days in patients with ICI combinations [183]). Furthermore, there is no pathognomonic clinical presentation, and differential diagnosis from other ICI-related GI toxicity can be extremely difficult. Celiac disease should be kept in mind in patients with persistent diarrhea treated with immunotherapy, especially those with serial negative colon biopsies, particularly if serum anti-tissue transglutaminase IgA (tTG-IgA) antibodies are present, due to their high sensitivity and specificity [183]. Duodenal biopsy is usually required to confirm the diagnosis, which typically demonstrates villous blunting, intraepithelial lymphocytosis, active neutrophilic duodenitis, or surface erosion and ulceration. Biopsy is also needed to rule out other causes of villous atrophy, such as parasitic infections (e.g., *Giardia lamblia*), autoimmune or drug-induced enteropathies, or eosinophilic gastroente-ritis. Intestinal lymphoma, Crohn’s disease, tropical sprue, and Whipple disease must also be taken into account [183].

As in non-ICI related CeD, conservative management with a gluten-free diet is the main therapeutic measure in ICI-celiac disease, with a generally good response; however, in resistant cases, systemic corticosteroids should be considered [182].

#### 2.5.2. Gastritis

Upper gastrointestinal tract adverse events, particularly regarding stomach or duodenum, were rarely initially described with ICI treatment, but with the widespread use of immunotherapy, several cases of gastritis have been reported, either associated with colitis or as isolated forms. Data from a literature review from the last 20 years [185] identifies 25 gastritis case reports, mostly secondary to the use of anti PD-1 antibodies or ICI combinations. The time to onset is highly variable, ranging from 2 weeks to more than 3 years, and the symptoms more frequently described comprise epigastric pain (60%), followed by anorexia (36%), nausea (32%), and vomiting (32%). The clinical severity, according to the Common Terminology Criteria for Adverse Events (CTCAE), differs in reports, with most of cases being grade 3 and requiring high-dose corticosteroids or a combination with other immunosuppressive drugs.

Besides clinical suspicion, gastroscopy with mucosal biopsies remains the gold stan-dard technique for diagnosis. Endoscopic typical findings range from mucosal erythema and ulcers to even necrosis [186]. Pathological findings that can help to distinguish ICI-related gastritis include chronic activitivywith increased intraepithelial lymphocytes [187] and significantly higher rates of apoptosis compared with non-ICI related gastritis, potentially identifiable with anti-Caspase 3 immunohistochemistry [188]. Pathologists should always rule out the presence of *Helicobacter pylori* (by Giemsa staining, for example). Nonetheless, to date, there are no ICI-related gastritis histological classifications, so a correlation between clinical history, time course, histological findings and the absence of other gastric damaging agents such as NSAIDs, remains key to differentiate an immune-related disease from the other causes [188].

In patients receiving immunosuppressive drugs for ICI-related gastritis, symptoms recurrence or exacerbation have been described due to reactivation of opportunistic pathogens such as CMV, *Pneumocystis jiroveci*, EBV, or even *Strongyloides stercoralis* hyperinfestation [189,190] with life-threatening complications [191]. The highest clinical suspicion is warranted in such cases due to the serious clinical implications and the potential misdiagnosis as gastritis-related symptom exacerbation. Endoscopic/histological evaluation is necessary for proper diagnosis and treatment.

#### 2.5.3. Cholangitis

Few cases of cholangitis have been reported among ICI-related hepatobiliary disorders [179]. A clinical picture of ICI-related cholangitis can simulate IgG4-related sclerosing cholangitis or primary sclerosing cholangitis, and is a result of bile duct dilation and wall-thickening after treatment initiation [179]. The time to onset oscillates from super acute presentations after first treatment administration to more than 11 months after initiation. As in non-ICI related disease, cholangitis can classically manifest with Charcot’s triad, as fever with chills, right upper abdominal pain, and jaundice, sometimes associated with hypotension and altered mental status (Reynold’s pentad) [192,193,194,195,196]. Rarely, routine blood analysis can evidence asymptomatic cases [197].

The first test usually performed upon clinical suspicion is a blood analysis demonstrating pure or mixed cholestasis. Alkaline phosphatase (ALP) and gamma glutamyl transpeptidase (GTP) are usually the most altered, with hyperbilirubinemia being a late finding; thus, significant elevations are suggestive of advanced disease [198]. De Ritis ratio (alanine transferase to ALP ratio) may assist in differential diagnosis, with values < 2 and > 5 favoring hepatitis and cholangitis, respectively [199]. Albeit seldom-mentioned in ICI-related cholangitis reports, hypercholesterolemia occurs in 75–95% of primary biliary cholangitis. Consequently, lipid alterations may be found along the disease course [200].

Screening for viral hepatitis, CMV, EBV, and other hepatotropic viruses is mandatory. Likewise, a comprehensive liver autoimmunity panel including ANA, antimitochondrial and smooth muscle antibodies, as well as IgG4 levels, is needed for distinguishing from other etiologies. Contrast-enhanced CT and MRI are useful, and usually show thickening of the extrahepatic and intrahepatic bile ducts [179] or even the gallbladder [201] without any obstructive cause. Endoscopic ultrasound (EUS) may demonstrate diffuse enlargement of the extrahepatic bile ducts [192]. A definitive diagnosis requires histologic confirmation by liver biopsy or biliary biopsies through endoscopic retrograde cholangiopancreatography (ERCP). Histopathological analysis on ICI-related cholangitis may reveal T-cells infiltrating bile ducts, with a predominance of CD8^+^ over CD4^+^ T cells. Kawakami et al. [192] proposed some ICI-related cholangitis characteristics including radiologic/endoscopic findings, laboratory abnormalities, as well as clinical and histological criteria, based on 3 NSCLC patients in the context of nivolumab-related cholangitis.

Ursodeoxycholic acid and immunosuppressive agents have been used for the treatment of ICI-related cholangitis, with reports of liver function improvement with drug discontinuation [195] and a corticosteroids course [196]. Notwithstanding, the outcome is usually poor, even with proper treatment [201].

### 2.6. Cardiac Immune-Related Adverse Events

Cardiovascular toxicity associated to ICI is less known due to its challenging diagnosis, variable clinical presentation, and overlapping features between entities. Occasio-nally, an overlooked cardiovascular toxicity may lead to fatal outcomes, highlighting the importance of prompt detection, monitoring and careful treatment.

Myocarditis is an inflammatory disease of the cardiac muscle (myocardium) that can develop into heart failure, dilated cardiomyopathy, and even sudden death, and may present in more atypical forms, such as rhythm disorders or Takotsubo-like syndrome [202]. Immune-related myocarditis occurs at a variable frequency, between 0.27% [203] and 1.14% [204] of patients, and has been widely described in the last few years [205,206]. Zimmer et al. also reported cases of ICI-related hypertension, symptomatic sinus tachycardia and angina pectoris, all of them resolving after treatment discontinuation [16]. Here, we focus on some of the more uncommon described adverse events.

#### Pericardial Disease

ICI-associated pericardial toxicity may present as cardiac tamponade, pericarditis, or pericardial effusion [207,208,209,210]. No systematic review has been published to date, but Salem et al. reported an incidence of 0.3%, being more prevalent with ICI combinations than monotherapy (0.16–0.33%), with a median onset of 30 days. Conversely, it has been observed that the use of corticosteroids prior to the initiation of ICI increased the risk of subsequent pericardial disease [206]. Gong et al. estimated a fourfold increase in the risk of pericardial disease among patients on ICI treatment compared to controls [211]. To date, no underlying mechanism on ICI-related pericardial disease has been proposed, perhaps due to its low frequency and lack of research.

Pericarditis usually presents with distinctive chest pain, although it can be complicated by pericardial effusion and even life-threatening cardiac tamponade. It is characterized by the presence of a new pericardial effusion on echocardiography; ECG changes with typical findings, such as PR segment depression and widespread saddle-shaped ST elevation; and is sometimes accompanied by arrhythmias, elevated troponin, and evidence of active pericardial inflammation on cardiac MRI or cardiac ^18^F-FDG PET/CT [212,213].

Withdrawal of ICI treatment remains crucial to avoid further fatal complications. To date, no management guideline has been published; the current practice is to initiate corticosteroids if there is an AE grade 2–4 pericardial disease. Subsequent tapering can be made after evaluation by a multidisciplinary medical team. Colchicine and NSAIDs could be administered as supportive therapy based on non–ICI-associated pericarditis guidelines. Urgent pericardiocentesis and hemodynamic support may be needed in case of cardiac tamponade [212,213,214].

Mortality rate is around 21%, and it has been described that patients who developed ICI-related pericardial disease have a trend for increased all-cause mortality compared to patients with no pericardial events [211].

### 2.7. Urologic Immune-Related Adverse Events

ICI-related urinary system studies are usually focused on kidney dysfunctions, including nephritis and acute kidney injury (AKI) that vary between 9.9–29% [215]. Acute tubule interstitial nephritis is the dominant pattern of ICI-induced AKI [216,217], and has been reported with an incidence as high as 6.7% [218]. Although initially overlooked, adverse events affecting the urinary bladder and tract have been reported more recently.

#### Non-Infectious Cystitis

Immune-related cystitis may be suspected in patients treated with ICI who develop symptoms of urinary tract irritation (such as dysuria, pollakiuria, or urinary urgency), leukocyturia or erythrocyturia in urine samples, and after infective etiologies have been ruled out by consecutive urinary cultures or antibiotic failure. We have found eight cases described in the literature [219] in patients receiving pembrolizumab, nivolumab, and to-ripalimab (a monoclonal anti PD-1 antibody approved in China [220]). Using data from the FDA adverse database, 19 reports were identified by He et al. [219].

Cystoscopy is mandatory to discard malignancy and for mucosal biopsy. Histopathology in ICI-related cystitis [221] may demonstrate nonspecific cystitis with inflammatory cell infiltration, interstitial edema, and epithelial abscission. Immunohistochemistry can help to identify CD8^+^ T-cell-restricted intracellular antigen (TIA)+ cytotoxic T cells in the epithelium, and lymphocyte vasculitis, otherwise not commonly found in ordinary nonspecific inflammation. The clinical response to corticosteroids supports the diagnosis in a compatible scenario.

The pathophysiology of ICI-cystitis remains unknown, yet some hypotheses have been postulated. On the one hand, PD-L1 expression has been correlated with interstitial non-infectious cystitis severity in a general population cohort [222]. An unknown antigen in the urothelium targeted by lymphocytes positive for TIA-1 and/or CD8 could explain overexpression after ICI exposure and self-damage [223]. Finally, the urinary microbiome may also be related to ICI response, and, consequently, dysbiosis could trigger an innate immune response over self-antigens [224].

Like treatment principles of other irAEs, immune-related cystitis is mainly treated with corticosteroids. Usually, urinary symptoms improve rapidly with therapy, and the duration of immunosuppressive treatment requirement is variable (from 3 weeks to 2 months). It was possible to restart ICI in five out the of seven patients described [219]. No information regarding outcomes have been found.

### 2.8. Ocular Immune Related Adverse Events

Eyes can also be affected by ICI-treatment, although its frequency has been less addressed until recently. Yet, ocular adverse events may particularly lead to a deterioration of the quality of life and may affect the compliance of patients on treatment. Non-granulomatous uveitis and dry eye syndrome are by far the most common ocular adverse events, presenting in a frequency ranging from 0.3 to 6% and 1.2 to 24%, respectively [225]. Optic neuritis [226], neuromyelitis optica with positive anti-aquaporin-4 antibodies [227,228,229,230], and even encephalomyeloneuritis [231] have also been reported. The average timing of irAEs from starting checkpoint inhibitor therapy is approximately 15 weeks [232].

#### Vogt–Koyanagi–Harada-Like Reaction

Vogt–Koyanagi–Harada disease (VKHd) is, on the contrary, a very rare and severe non-infectious, bilateral, granulomatous, posterior, or panuveitis associated with serous retinal detachments, vitritis, disc edema, and the development of a sunset glow fundus [233]. The disease is also associated to systemic symptoms, such as hearing loss, poliosis, skin sensitivity and vitiligo, cranial nerve palsies, and meningismus [234]. In all forms of the disease, there should be no history of ocular surgery or trauma, and no clinical or laboratory evidence suggestive of other ocular diseases [235]. VKHd presents clinically in four different phases: prodromal, acute uveitic, convalescent, and chronic recurrent. The spectrum of manifestations differs across these phases; hence, a high grade of suspicion is needed for prompt diagnosis and treatment.

An ICI-related Vogt–Koyanagi–Harada-like reaction is a very rare event, with a median time to onset of around 10 weeks (range, 4 to 18 weeks) after treatment initiation [218]. Most of the cases have been described in melanoma patients, which raises questions about the relation between melanoma and the classical altered immune response to melanocytes, possibly distorted in ICI-treated patients. A possibly confounding factor arises, as ICI are the mainstay of treatment for most melanoma patients, which may explain its higher frequency. Nonetheless, more recent reports have also described VKH-like reactions in ovarian and non-small cell lung cancer [181,222,223].

Slit-lamp examination may show bilateral granulomatous keratic precipitates and anterior chamber cells. Wavy retinal pigments, as well as serous retinal detachment, may be found on optical coherence tomography. Specific indocyanine green angiography usually reveals choroidal hyperfluorescence due to choroidal vascular leakage and hypofluo-rescent dark spots during the late phase [236]. Cerebrospinal fluid lymphocytic pleocytosis may be present in around 80% of cases, also with hyperproteinorhaquia and low glucose levels [233]. The diagnosis of a complete VKH is made by strictly fulfilling five diagnostic criteria, including no history of penetrating ocular trauma, along with physical characteristic findings [235]. Differential diagnosis may include other forms of granulomatous and non-granulomatous uveitis, including pars planitis, sympathetic ophthalmia, and HLA-B27(+) uveitis.

Topical, oral, or subconjunctivally-injected corticosteroids may be used for treatment, with most patients responding partially or completely [237,238]. Information regarding cancer outcomes is scarce, although some prolonged responses following the resolution of the iRAE have been reported [239].

## 3. Conclusions and Perspectives

Treatment with check-point inhibitors is nowadays the standard of care in a myriad of neoplasms in different stages and scenarios, as monotherapy or in combination with other agents. Given its unique mechanism of action of boosting the immune system response, restoring normal immunogenic clearance mechanisms, and enhancing the capacities of cytotoxic T cells, immunotherapy has brought a durable response in multiple tumor types, lighting the way for a growing proportion of long responders with chronically controlled diseases. Immunoncology is an area of intense clinical and translational research, and the pursuit of new and more reliable biomarkers—prognostic and predictive of response—is a matter of passionate investigation.

Along with these extraordinary results, immunotherapy has also introduced us to a completely new spectrum of adverse events, characterized by the paucity of manifestations and countless syndrome associations, variable onset time and, sometimes, the overlap with other cancer-related diseases. Since the first ICI approval, oncologists have become rapidly aware of the more frequent adverse events, and multidisciplinary approaches to these common situations are nowadays standardized. As the use of ICI spreads and indications exponentially increase in more tumor types and stages, along with longer patient survival, uncommon adverse events have been seen more frequently in clinical practice, encompassing new diagnostic challenges and requiring higher degrees of suspicion.

**Figure 2:**
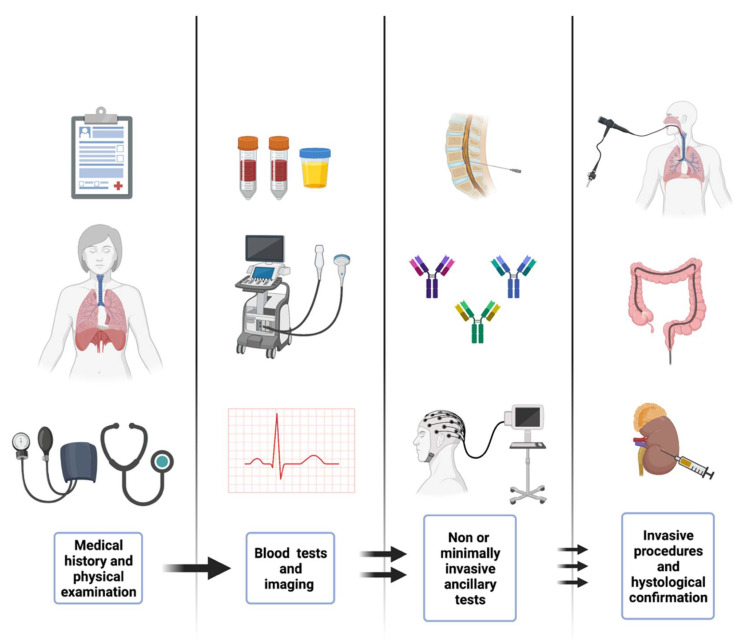
Proposed diagnostic workup for irAEs.

The diagnostic workup of common and, especially, uncommon irAEs requires careful physical examination, looking for the systemic manifestation of primarily organ-confined diseases, followed by basic blood and imaging tests. This basic workup may give the clinician an initial syndromic diagnosis, which may be thereafter definite by non-, minimally, and fully invasive procedures for histologically, serologically, or functionally confirmed diagnosis (see Figure 2).

Each step of this diagnostic workflow must be guided by clinical suspicion and carefully evaluated in a multidisciplinary team, which will translate to an earlier diagnosis, appropriate treatment, and, ultimately, to better outcomes.

This review addressed some of the most important and interesting irAEs, aiming to reunite the evidence published previously in clinical cases and retrospective reviews, allowing an increase in the awareness of uncommon irAEs, and an improvement in the access to information by all the specialists implicated in the diagnosis, treatment, and care of cancer patients treated with ICI. In the current scenario, the integration of biomarkers and new tools for the early diagnosis is eagerly awaited, since it will improve the diagnostic workout, guiding, in a near future, the therapeutic approach of these complex uncommon irAEs.

## Figures and Tables

**Table 1:** Summary of reported cases of ICI-related LEMS.

References	Age (y), Sex	Cancer Histology and Stage	ICI Drugs	Time to Onset	Findings	Treatment	Outcome
Nakatani et al. [35]	73; female	Stage IV non-small cell lung cancer	Nivolumab	20 weeks	Bilateral ptosis, limb weakness, photophobia, hyporeflexia, autonomic dysfunction. Positive anti-P/Q-type voltage-gated calcium channel (VGCC) antibodies (-ab)	Pyridostigmine; 3,4-diaminopyridine, low-dose corticosteroids. Initially restarted nivolumab, then definite discontinuation.	Progressive disease
Agrawal and Agrawal [36]	59; male	Stage IV non-small cell lung cancer	Nivolumab/ Ipilimumab	16 weeks	Gait disturbance, limb weakness, hyporeflexia No information regarding Abs status	Pyridostigmine; corticosteroids. ICI discontinuation	Progressive disease
Lee et al. [37]	80; male	Squamous and large cell neuroendocrine lung carcinoma	Pembrolizumab	10 months	Upper and lower limb weakness VGCC-ab not available	ICI discontinuation, corticosteroids, azathioprine	Maintained response for 12 months after ICI discontinuation
Duplaine et al. [38]	58; female	Extensive stage small cell lung carcinoma	Nivolumab	8 months	Ptosis, dysphagia. Proximal muscle weakness, hyporeflexia, autonomic dysfunction. Concomitant MG and LEMS with positive AChR-ab and anti-P/N-type VGCC-ab	Corticosteroids, anticholinesterase drugs, amifampridine, plasmapheresis, IVIG ICI discontinuation	Oligoprogressive brain disease treated with stereotactic surgery. Complete response up to 27 months after ICI discontinuation
Gill et al. [39]	58; female	Stage IV melanoma	Nivolumab	3 weeks	Diplopia, gait disturbance. Positive anti-P/Q-type VGCC-ab	3,4-diaminopyridine, prednisone, IVIG, rituximab	No disease recurrence after 24 months after ICI withdrawal
Kunii et al. [40]	74; male	Extensive stage small cell lung cancer	Atezolizumab	12 months	Upper and lower limbs weakness, fatigue. Slight gait disturbance and absent patellar reflexes. Positive anti-P/Q-type VGCC-ab	High-dose corticosteroids, IVIG, ICI discontinuation	Progressive disease

**Table 2:** Summary of diagnostic workup of neuromuscular irAEs.

	Electromyographic and Other Findings	Autoantibodies	Differential Diagnosis and Workup	Treatment
Myasthenia gravis	Muscle action potential decrement at baseline on low-rate repetitive stimulation [31]	AChR-Ab positive in up to 2/3 of patients. Anti-MuSK-Ab almost always negative [17]	Lambert–Eaton myasthenic syndrome, check for concurrent myopathies or myocarditis [31]	Pyridostigmine, high-dose IV corticosteroids, intravenous immunoglobulins (IVIG), and plasmapheresis [19,27,31]
Myopathies	Myopathic pattern with fibrillation and myopathic recruitment [42,43]	Anti-Ro/SSA, anti-DNAPK, anti-PM-Scl, anti-Scl70, anti-Jo-1, anti-MDA5, anti-TIF-1, anti-Mi-2, anti-NXP2 [47,48]	Check for concurrent myasthenia gravis or myocarditis [31]	High-dose IV corticosteroids, IVIG, plasmapheresis, infliximab [17,25,34,42,46,47,49,50]
Guillain–Barré Syndrome	EMG: Acute demyelinating polyradiculoneuropathy, prolonged distal latencies, low conduction velocities. CSF: mild pleocytosis with lymphocytic predominance (50%), hyperproteinorrhaquia, albumincytologic dissociation (44%) [54]	Anti-gangliosides antibodies occasionally positive [54]	Botulism, tick paralysis, intermediate syndrome in organophosphate poisoning, meningeal carcinomatosis, Lyme disease, West Nile virus flaccid paralysis. Stroke, brainstem metastases, and multiple sclerosis to be considered if total ophthalmoplegia is present [51,53,54,55]	IVIG and plasmapheresis. Some reports of response to high-dose corticosteroids [17,57,58]

**Table 3:** Bone marrow findings in ICI-related cytopenias [59,65,66,75,76,83].

Immune-Related Cytopenia	Bone Marrow Findings
Immune thrombocytopenia	Moderate hypercellularity and increased megakaryocytes
Hemolytic anemia (AIHA)	Not mandatory for diagnosis. Consider if an underlying cause is deemed possible. Erythroid hyperplasia may be found
Pure red cell aplasia	Erythroid hypoplasia accompanied by a granulocytic hyperplasia and adequate numbers of mature-appearing megakaryocytes in an otherwise normocellular bone marrow
Immune neutropenia	Blockage in granulocyte maturation (44%), granulocytic lineage hypoplasia (22%), or a near normal granulocytic lineage and bone marrow smear in up to 33%
Aplastic anemia	Marked hypocellularity (less than 20%) without blasts, and no reticulin fibrosis

**Table 4:** Rare cutaneous immune-related adverse events reported.

Steven Johnson syndrome [145,146]
Toxic epidermal necrolysis [146,147]
Drug reaction with eosinophilia and systemic symptoms [148,149,150]
Erythema nodosum-like panniculitis [151,152]
Grover’s disease [153,154,155]
Sjögren’s syndrome [156]
Scleroderma reaction [157]
Urticaria [158]
Eruptive keratoacanthomas [159]
Neutrophilic dermatoses (Sweet’s syndrome, pyoderma gangrenosum) [160,161,162,163]
Necrotizing vasculitis [164]
